# Laser-Deposited Beta Type Ti-42Nb Alloy with Anisotropic Mechanical Properties for Pioneering Biomedical Implants with a Very Low Elastic Modulus

**DOI:** 10.3390/ma15207172

**Published:** 2022-10-14

**Authors:** Felipe Arias-González, Alejandra Rodríguez-Contreras, Miquel Punset, José María Manero, Óscar Barro, Mónica Fernández-Arias, Fernando Lusquiños, Javier Gil, Juan Pou

**Affiliations:** 1 LaserOn Research Group, CINTECX, School of Engineering, Universidade de Vigo (UVIGO), Lagoas Marcosende, 36310 Vigo, Spain; 2 Biomaterials, Biomechanics and Tissue Engineering Group, Materials Science and Engineering Department, and Research Center for Biomedical Engineering, Universitat Politècnica de Catalunya (UPC), 08019 Barcelona, Spain; 3 Institut de Recerca Sant Joan de Déu (IRSJD), 08034 Barcelona, Spain; 4 Barcelona Research Center in Multiscale Science and Engineering, Universitat Politècnica de Catalunya (UPC), 08019 Barcelona, Spain; 5 UPC Innovation and Technology Center (CIT-UPC), Universitat Politècnica de Catalunya (UPC), 08034 Barcelona, Spain; 6 Galicia Sur Health Research Institute (IIS Galicia Sur), SERGAS-UVIGO, 36312 Vigo, Spain; 7 School of Dentistry, Universitat Internacional de Catalunya (UIC), 08195 Sant Cugat del Vallès, Spain

**Keywords:** microstructure, crystallographic texture, Young’s modulus, laser-directed energy deposition, titanium alloys, cytocompatibility

## Abstract

Present commercial titanium alloy implants have an elastic modulus higher than 100 GPa, whereas that of the cortical bone is much smaller (17–28 GPa). This elastic modulus mismatch produces a stress shielding effect and the resorption of the bone surrounding the implant. In the present work, a <100> fiber texture is developed in β type Ti-42Nb (wt%) alloy ingots generated by laser-directed energy deposition (LDED) in order to achieve anisotropic mechanical properties. In addition, we demonstrate that laser-deposited β type Ti-42Nb alloy ingots with an intense <100> fiber texture exhibit a very low elastic modulus in the building direction (E_z_ < 50 GPa) and high yield (σ_0.2z_ > 700 MPa) and tensile (UTS_z_ > 700 MPa) strengths. Laser-deposited Ti-42Nb alloy enhances the osteoinductive effect, promoting the adhesion, proliferation, and spreading of human osteoblast-like cells. Hence, we propose that laser-deposited β type Ti-42Nb alloy is a potentially promising candidate for the manufacturing of pioneering biomedical implants with a very low elastic modulus that can suppress stress shielding.

## 1. Introduction

Metallic materials and alloys are commonly employed in biomedical applications, primarily for the restoration of damaged hard tissue [1,2]. Commercially pure titanium (cp-Ti) and titanium alloys, Co-Cr alloys, and even stainless steels are widely applied in this field due to their superior strength and toughness. Their mechanical properties are the most essential safety requirements for a biomaterial under load-bearing conditions [1,2,3]. Nowadays, they cannot be replaced by polymers or ceramics [3].

Commercially pure titanium (cp-Ti) and Ti-6Al-4V are thought of as the most appropriate metallic materials for orthopedic and dental applications because of their excellent properties, such as good biocompatibility, superb corrosion resistance, toughness, and a low elastic modulus [1,2,3,4,5,6,7]. However, cp-Ti and Ti-6Al-4V exhibit some weaknesses: the wear resistance and strength of cp-Ti is not high enough for some biomedical applications [3]; and Ti-6Al-4V has excellent strength, but the release of toxic V and Al ions in the human body can originate long-term health complications [1,2,3,4,5,6,7,8].

Furthermore, the elastic modulus of cortical bone (17–28 GPa) is much lower than that of cp-Ti (102.7–104.1 GPa) and Ti-6Al-4V (101–114 GPa) [7]. Since the elastic modulus of the surrounding bone is lower than that of the biomedical implant, a phenomenon called stress shielding can happen [1,3,4,5,6,8,9,10]. The implant takes up the applied stresses instead of the bone, thus bone regeneration is inhibited while the resorption of the bone by the body increases. As a result, some issues such as the loosening of the implant or weakening of the bone can occur [1,3,4,5,6,8,9,10].

In recent years, new biomedical β type titanium alloys have been developed from elements with low toxicity and good biocompatibility, such as Ta, Nb, or Zr [1,3,4,5,6,7,8,9]. These alloys stand out because of their mechanical properties (great strength and low elastic modulus) and excellent biocompatibility and corrosion resistance [11,12]. These β type Ti alloys are produced by adding enough amounts of β-stabilizers (e.g., Ta or Nb) and quenching to retain the β-phase, while the formation of other phases is avoided [11,12].

Binary Ti-Nb alloys are promising non-toxic materials for biomedical implants because of their strong corrosion resistance and excellent biocompatibility [13,14,15,16,17,18,19]. The critical concentration of Nb required to preserve the β-phase at room temperature is 36 wt%, so the metastable β-phase is retained at higher concentrations [20]. It is known that the elastic modulus of Ti-Nb alloys presents an absolute minimum (~62 GPa) at an Nb content of 40–45 wt% [15,20,21]. This elastic modulus is lower than that of cp-Ti (102.7–104.1 GPa) or Ti-6Al-4V alloy (101–114 GPa), but still considerably higher than that of cortical bone (17–28 GPa) [7]. Therefore, an extra decrease of the elastic modulus is still required.

A new proposal to reduce the elastic modulus of biocompatible β type Ti alloys’ implants is based on controlling the distribution of crystallographic orientations of the grains (crystallographic texture). It is known that the elastic modulus of β type Ti alloys’ single crystals has a strong crystallographic orientation dependence. In fact, the elastic modulus of β type Ti_70_Nb_30_ (at%) alloy, equivalent to Ti-45Nb (wt%), shows pronounced anisotropy, with the highest value along orientation <111> (E_111_ = 91 GPa) and the lowest value along orientation <100> (E_100_ = 39.5 GPa) [22]. The same dependence has been demonstrated for other biocompatible β type Ti alloys [23,24,25,26,27]. Consequently, the elastic modulus of β type Ti alloys could be decreased by controlling their crystallographic texture. Nevertheless, in practice, one of the challenges of generating biomedical implants with controlled crystallographic texture lies in the need for new processes to produce them [28,29].

In 2017, Ishimoto et al. successfully generated two types of crystallographic textures in β type Ti-15Mo-5Zr-3Al (wt%) alloy components additively manufactured by selective laser melting (SLM), also known as laser powder bed fusion (LPBF) [28]. Different scanning strategies gave rise to different crystallographic textures with preferential orientations of <100> and <110> along the building direction. The generated material presents a low elastic modulus of 68.7 ± 0.9 GPa, which could allow the development of implants that can suppress stress shielding. In 2022, Pilz et al. also employed LPBF to generate β type Ti-42Nb (wt%) alloy samples. Utilizing an unconventional top hat laser configuration, a microstructure with an intense <100> texture parallel to the building direction was generated [30]. An elastic modulus as low as 44 GPa was measured along this direction.

In 2018, Shinohara et al. successfully developed the <100> fiber texture in cold-groove-rolled Ti-Mo-Al-Zr alloy wires [29]. It was the first report on the generation of the <100> fiber texture as the main component in β type Ti alloy rods or wires. The elastic modulus of samples along the rolling direction decreased with an increase in the CSA reduction rate. This effect was explained by the formation of the <100> fiber texture.

In the present work, a <100> fiber texture was developed in β type Ti-42Nb (wt%) alloy ingots generated by means of laser-directed energy deposition (LDED) in order to achieve anisotropic mechanical properties and a very low elastic modulus. Laser-directed energy deposition (LDED) is an additive manufacturing (AM) method for producing parts by delivering energy and materials simultaneously [31,32,33]. A laser beam is employed as a heat source to melt material that is selectively deposited on a substrate, where it solidifies. This manufacturing process is more suitable to produce larger components faster than SLM because of their high deposition rates [31]. Nevertheless, the high deposition rates require large molten pools resulting in rough beaded surfaces. Machining could be required to achieve the final functional geometry. LDED can be applied to any material that can be melted and supplied in the form of wire or particles, such as metals and some ceramics [33,34,35,36,37,38,39,40,41].

LDED has already been explored to generate parts of cp-Ti and titanium alloys [31,32,33,34], such as improved cp-Ti ingots for dental prosthetic applications [36]; porous Ti parts [42,43]; Ti-6Al-4V scaffolds [44]; or biocompatible β type Ti-Nb [37,45,46,47], and Ti-Zr-Nb [37], and Ti-35Nb-7Zr-5Ta (wt%) [48,49,50]. Laser-deposited β type Ti alloys consist of a single metastable β-phase because of the high cooling speed achieved during the process [37,45,46,48,49,50]. It also has been reported that β type Ti alloys generated by LDED exhibit vertical columnar β grains [37,46]. Moreover, these grains present a relatively intense <100> fiber texture, with a considerably great quantity of grains of the β-phase aligning one of their <100> axes as nearly normal to the substrate or parallel to the building direction [46,48].

This background suggests that LDED is a feasible technique to additively manufacture biocompatible β type Ti alloy ingots with an intense <100> fiber texture. These ingots should exhibit anisotropic mechanical properties because of their crystallographic texture and, particularly, a very low elastic modulus along the building direction. In the present work, we additively manufactured biocompatible β type Ti-42Nb (wt%) alloy by means of LDED to produce ingots with a <100> fiber texture in order to evaluate the theses hypotheses. Microstructure, phase composition, crystallographic texture, and mechanical properties were analyzed by means of scanning electron microscopy (SEM), X-ray diffraction (XRD), electron backscatter diffraction (EBSD), and uniaxial tensile tests, respectively. To our knowledge, this is the first report studying the elastic modulus in different directions of laser-deposited biocompatible β type Ti alloy ingots. Finally, in vitro cytocompatibility was assessed with human cells.

## 2. Materials and Methods

### 2.1. Generation of Ti-42Nb Specimens by Laser-Directed Energy Deposition (LDED)

**Figure 1:**
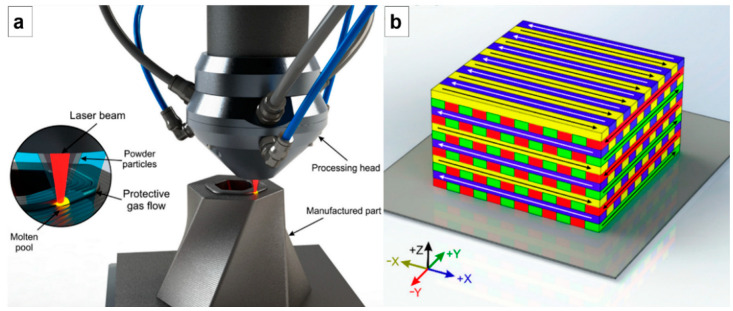
(**a**) Sketch of laser-directed energy deposition experimental configuration. (**b**) Tridimensional illustration of the scanning strategy employed to generate the laser-deposited specimens [36].

The laser-directed energy deposition (LDED) technique was employed to produce Ti-42Nb specimens. Figure 1a displays an outline of the experimental configuration. The system was composed by a High Power Diode Laser (HPDL) with a maximum output power of 1600 W and a wavelength between 915 and 976 nm (DILAS Diodenlaser GmbH, Mainz, Germany), a laser head with coaxial powder injection, a commercial pneumatic powder feeder (Oerlikon Metco Europe GmbH, Pfäffikon, Switzerland), and a CNC controlled positioning system. The manufacturing process was carried out in a controlled-ambient inert chamber that employed argon as a protective atmosphere (O_2_ < 50 ppm).

**Figure 2:**
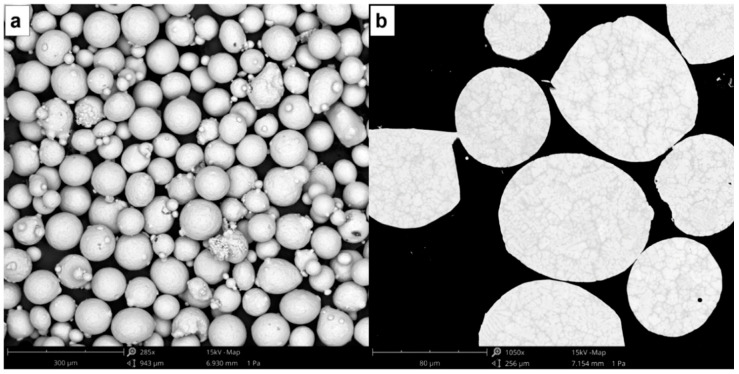
SEM micrographs of spherical pre-alloyed Ti-42Nb powder used as precursor material to generate the specimens: (**a**) particle size and morphology, and (**b**) microstructure.

To obtain the testing specimens, flat plates of commercially pure titanium (cp-Ti grade 2) with dimensions of 100 × 50 × 10 mm^3^ were used as substrates. The laser beam from the diode laser was focused on the surface of these titanium plates by means of a lens with a diameter of 50 mm and a focal length of 250 mm, which provided a focal spot of approximately 3 mm. The testing specimens were obtained with the following processing conditions: laser power of 1000 W that gave a laser irradiance of ~140 W/mm^2^, 2.2 g/min of Ti-42Nb powder mass flow, and a processing speed of 6 mm/s. A layer-by-layer deposition process was carried out to obtain three-dimensional specimens, following the strategy shown in Figure 1b. The beads were deposited following a round trip path, so that at the end of a bead, there was a 90° rotation in the path of the laser beam. Once a layer was finished, a 45° rotation occurred so that the next layer was deposited perpendicular to the direction in which the previous layer was deposited. The distance between each clad strip was established at 2.5 mm, which represents an overlap ratio of 20%. Each layer was about 0.33 mm high. Spherical pre-alloyed Ti-42Nb powder with a particle size between 63 and 105 µm (AMtrinsic^®^ Ti-42Nb 63–105 µm, Taniobis GmbH, Goslar, Germany) was used as the precursor material (Figure 2).

**Figure 3:**
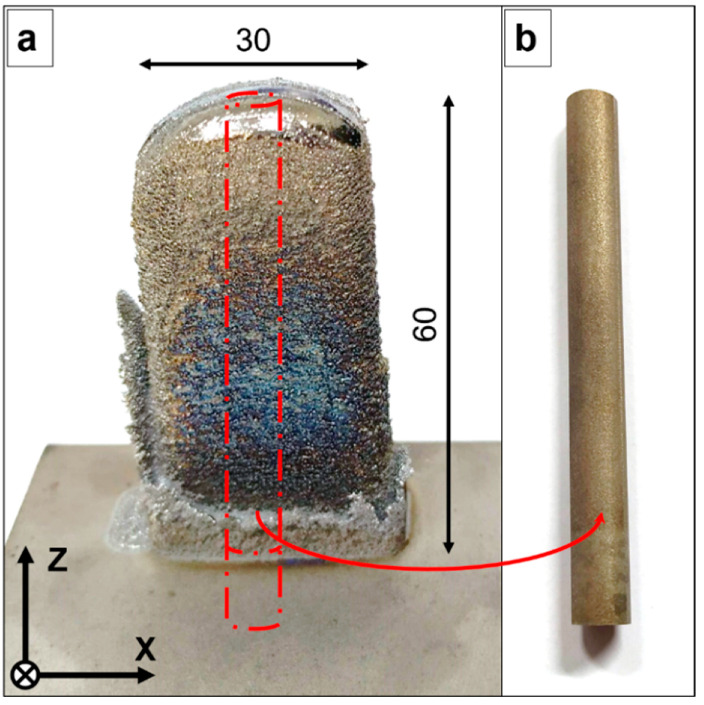
(**a**) Laser-deposited Ti-42Nb vertical specimen. (**b**) Vertical cylindrical sample. All dimensions are shown in mm.

**Figure 4:**
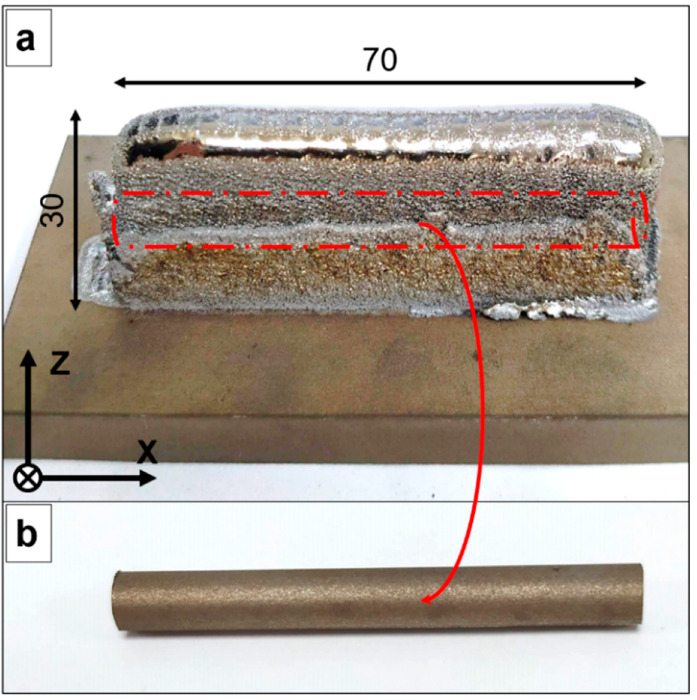
(**a**) Laser-deposited Ti-42Nb horizontal specimen. (**b**) Horizontal cylindrical sample. All dimensions are shown in mm.

Two types of laser-deposited geometries were generated (Figure 3a and Figure 4a): vertical specimens (12.5 mm × 30 mm × 60 mm), and horizontal specimens (12.5 mm × 70 mm × 30 mm). After that, wire electrical discharge machining (WEDM) was employed to obtain vertical cylindrical samples and horizontal cylindrical samples (Ø 7 mm) from the vertical and horizontal specimens, respectively (Figure 3b and Figure 4b).

### 2.2. Microstructure and Crystallographic Texture Characterization

The cylindrical samples were cut into discs, embedded in resin, and polished to study the XY cross-section through z = 10, 30, and 50 mm (measured from the top of the substrate) on the vertical samples, and the YZ cross-section on the horizontal samples. The microstructure was analyzed by scanning electron microscopy (SEM, Phenom XL, Thermo Fisher Scientific, Waltham, MA, USA). The determination of the phases present in the samples built by LDED was carried out using a Bruker D8 Advance X-ray diffractometer (Bruker, Billerica, MA, USA). For this, the monochromatic radiation from a copper target was used. The CuKα radiation of 1.54 Å wavelength was collimated and directed onto the testing samples. Data were collected at 2θ from 20 to 110° with a step size of 0.02°. Diffraction patterns were acquired from the surface of the LDED built samples that were previously polished, and from the spherical pre-alloyed Ti-42Nb particles to identify their constituent phases. The crystallographic texture of the samples was observed by electron backscatter diffraction (EBSD, JEOL JSM-7001F, JEOL Ltd., Akishima, Japan) and analyzed using the ATEX software [51].

### 2.3. Mechanical Characterization

**Figure 5:**
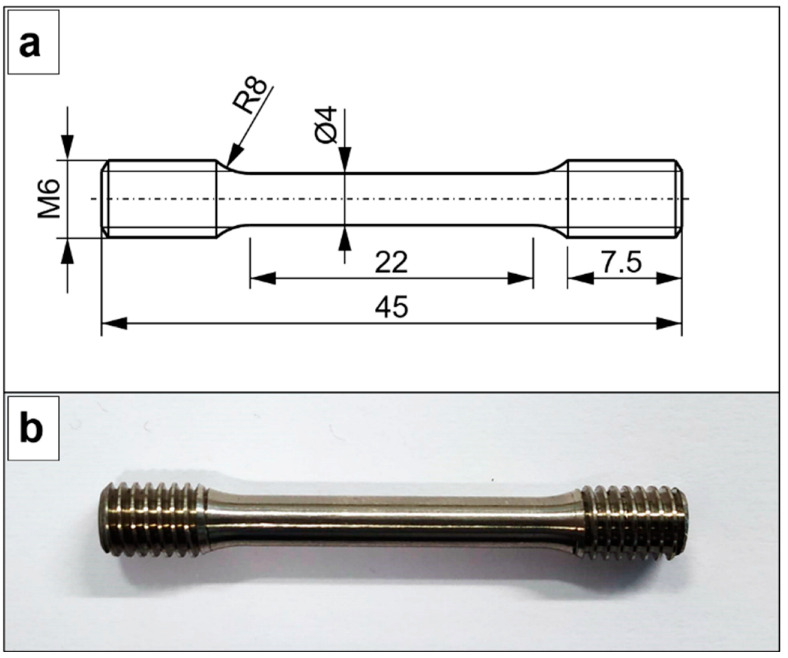
(**a**) Geometrical dimensions of the specimens for tensile test. (**b**) Specimen for the tensile test. All dimensions are shown in mm.

Three cylindrical vertical samples and three cylindrical horizontal samples were machined into specimens for the tensile test (Figure 5). The original gauge length was 20 mm. Uniaxial tensile tests were carried out according to the ISO 6892-1:2020 [52] standard using a Zwick Z100 universal machine (Zwick Roell Group, Ulm, Germany). The values of the elastic modulus (E), yield strength (σ_0.2_), ultimate tensile strength (UTS), and elongation at break (ε_b_) were obtained for each specimen.

### 2.4. Cytocompatibility Assessing

#### 2.4.1. Contact Angle

The contact angle with water and diiodomethane was measured for the determination of the surface energy. The sessile drop method was used to estimate the contact angles in the static regime. These measurements were carried out by means of an OCA15 Plus goniometer from DataPhysics Instruments (Filderstadt, Germany). Droplets of 3 μL were used to evaluate the surface wettability in different points of the sample. All measurements were carried out using a dose of 1 μL/s at room temperature. The model developed by Owens, Wendt, Rabel, and Kaelble, better known as the OWRK method, was used to calculate the surface free energy of the samples as well as the dispersive and polar components.

#### 2.4.2. Sample Sterilization and Cell Culture

Commercially pure Ti grade 2 discs, used as the control, and laser-deposited Ti-42Nb discs were sterilized by repeatedly washing with 70% ethanol solution: each bath took 5 min and was repeated 3 times; following a 30 min exposure to UV radiation.

Biocompatibility of the LDED built materials was evaluated in vitro using human osteoblast-like cells SaOS-2 (cell line ATCC HTB-85, Manassas, VA, USA). McCoy’s 5A medium (Sigma–Aldrich, St. Louis, MO, USA) was used to culture the cells. Fetal bovine serum (FBS) diluted at 10% together with 1% penicillin/streptomycin antibiotics (50 U/mL and 50 μg/mL, respectively) were used to supplement the culture medium. Cell cultures were carried out at a temperature of 37 °C in 5% (*v*/*v*) CO_2_ and humidified atmosphere.

Falcon 12-well clear multiwall plates were used to perform the tests with 20,000 cells/well. Discs of both laser-deposited Ti-42Nb samples and cp-Ti grade 2 control were placed in the wells as substrates for the cell cultures. The cell adhesion studies were performed on cultures running for 4 h, whereas cell proliferation studies lasted 3, 7, and 14 days.

Afterward, paraformaldehyde (PFA) at 4% in phosphate-buffered saline (PBS) was used to fix the cells. Cell fixing took 30 min and was carried out at a temperature of 4 °C. After the PFA solution immersion, the samples were washed three times with PBS at a pH of 7.2–7.4 (each bath took 5 min) and kept at a temperature of 4 °C for analysis.

#### 2.4.3. Cell Preparation for Observation in Field Emission Scanning Electron Microscopy

In total, 4% Paraformaldehyde solution in PBS was used to fix the cells cultured on laser-deposited Ti-42Nb samples and the cp-Ti grade 2 control. The treatment took 30 min and was carried out at a temperature of 4 °C. Samples were washed 3 times with PBS, taking 5 min each bath. A graded sequence of aqueous ethanol (30–100%) was used to dehydrate the cells. Afterward, cells were dried at room temperature for 24 h. The cell examination was carried out by a field emission scanning electron microscope (FESEM, Carl Zeiss Neon 40 Crossbeam, Carl Zeiss AG, Oberkochen, Germany) operating at a voltage of 2 kV. For quantitative cell counting, ten images were randomly selected at 300× magnification from the surface of four samples. The cell area was determined by an image analysis using a Java-based image processor (ImageJ).

## 3. Results

**Figure 6:**
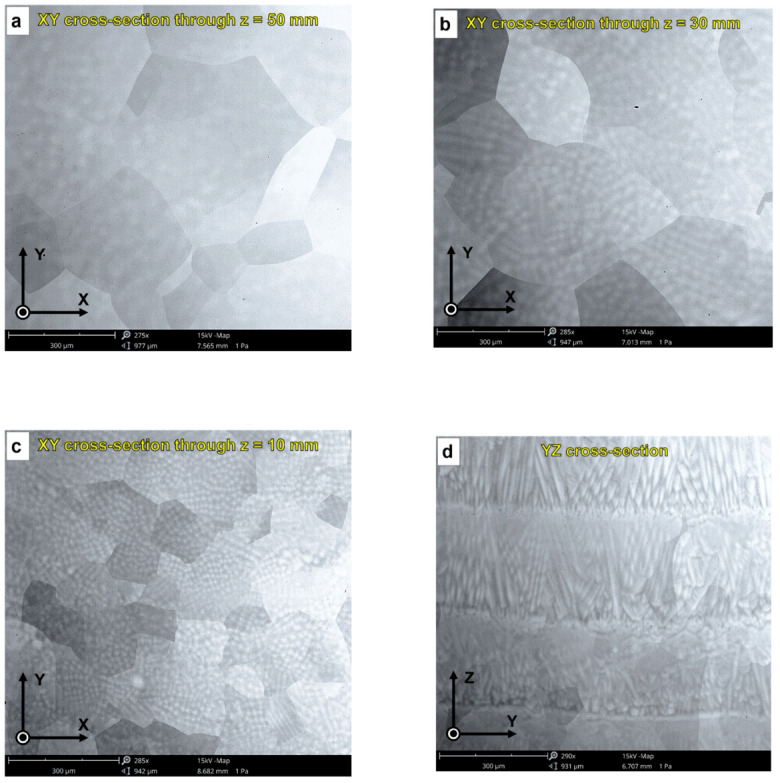
SEM micrographs of laser-deposited Ti-42Nb samples: (**a**) XY cross-section through z = 50 mm, (**b**) XY cross-section through z = 30 mm, (**c**) XY cross-section through z = 10 mm, and (**d**) YZ cross-section.

We successfully fabricated dense β type Ti-42Nb alloy ingots via LDED by appropriately tuning the process parameters. The microstructure of the Ti-42Nb samples is displayed in the SEM micrographs in Figure 6. The analysis was performed on the XY cross-section at different heights (measured from the top of the substrate), and on the YZ cross-section. Coarser grains were observed on the top of the specimen, at z = 50 mm and 30 mm (Figure 6a and Figure 6b, respectively) than on the bottom, at z = 10 mm (Figure 6c). The samples exhibited a dendritic segregation on the bottom that tended to disappear on the middle and top. These dendrites were aligned parallel to the building direction (Z-axis), which indicates that grains grow epitaxially from the parent grains in the molten pool as a consequence of the high thermal gradient (Figure 6d). This epitaxial columnar grain growth is a microstructural feature commonly observed in laser-deposited titanium alloys [32,34,37,44,45,46,48].

**Figure 7:**
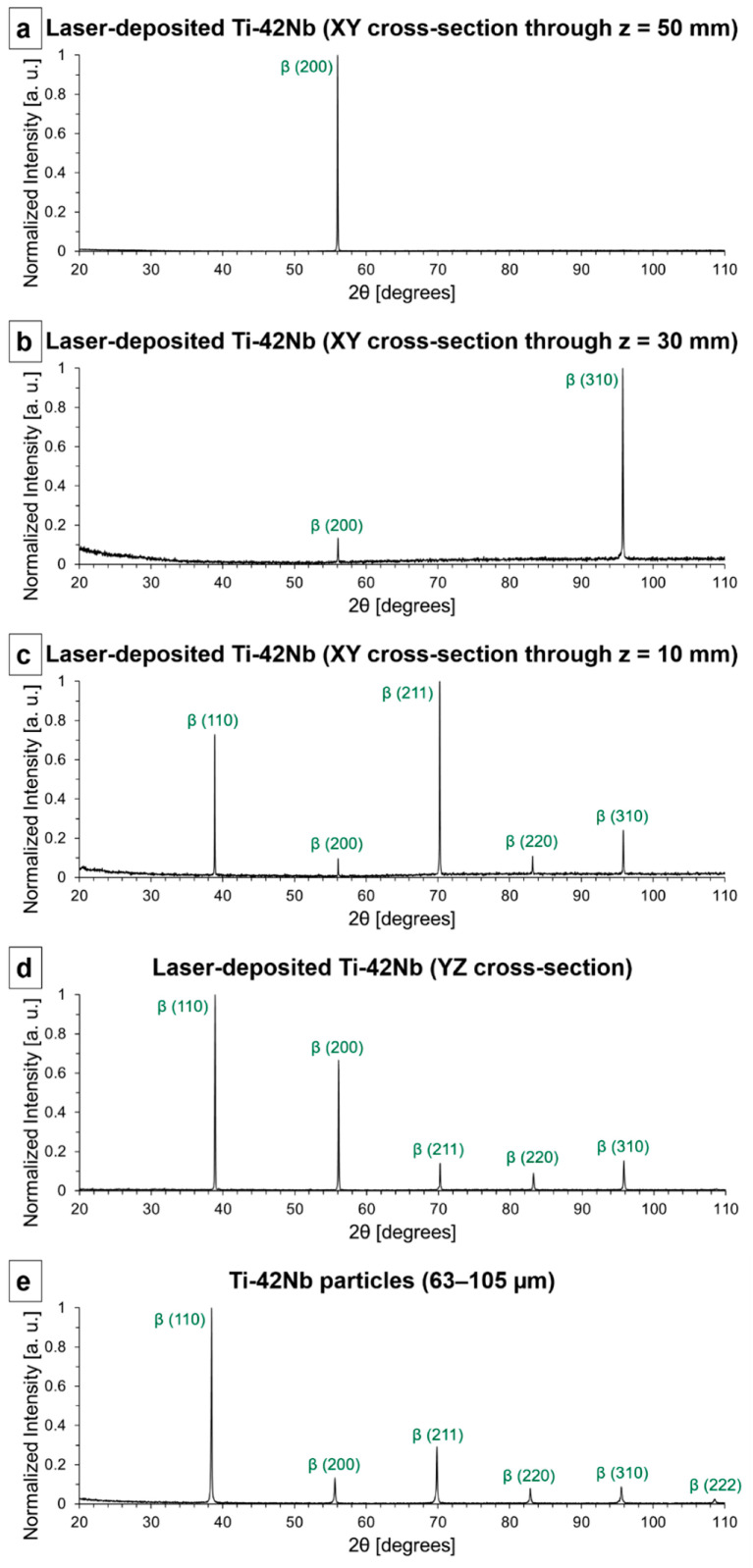
X-ray diffractograms: (**a**) laser-deposited Ti-42Nb (XY cross-section through z = 50 mm), (**b**) laser-deposited Ti-42Nb (XY cross-section through z = 30 mm), (**c**) laser-deposited Ti-42Nb (XY cross-section through z = 10 mm), (**d**) YZ cross-section, and (**e**) spherical pre-alloyed Ti-42Nb particles (63–105 µm).

X-ray diffraction (XRD) studies were carried out on the same samples studied by SEM to confirm that their composition was primarily single β-phase. The diffractograms are shown in Figure 7. A sample of the powder employed as a precursor material, spherical pre-alloyed Ti-42Nb particles (63–105 µm), has been studied by XRD as well (Figure 7e). The Ti-42Nb powder was formed by randomly oriented single β-phase crystallites and its diffractogram has been employed as a reference pattern of a β-phase polycrystalline material with random crystallographic texture. It can be observed that some peaks were missed in the diffraction patterns of the samples generated by LDED (Figure 7a–d), and there were discrepancies in the relative intensity of the peaks in comparison to the reference pattern (Figure 7e). These variations were caused by two factors. Firstly, the microstructure of the laser-deposited samples was composed of coarse grains and the area studied by XRD did not comprehend a statistically relevant number of grains that contributed to the diffraction pattern. Secondly, the crystallographic texture of the laser-deposited Ti-42Nb was not random.

**Figure 8:**
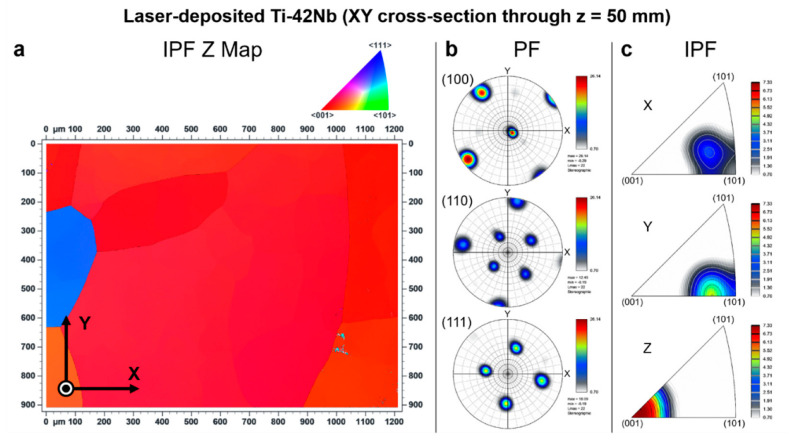
Electron backscatter diffraction (EBSD) results at XY cross-section through z = 50 mm: (**a**) inverse pole figure Z map (IPF Z Map), (**b**) pole figures (PF), and (**c**) inverse pole figures (IPF).

**Figure 9:**
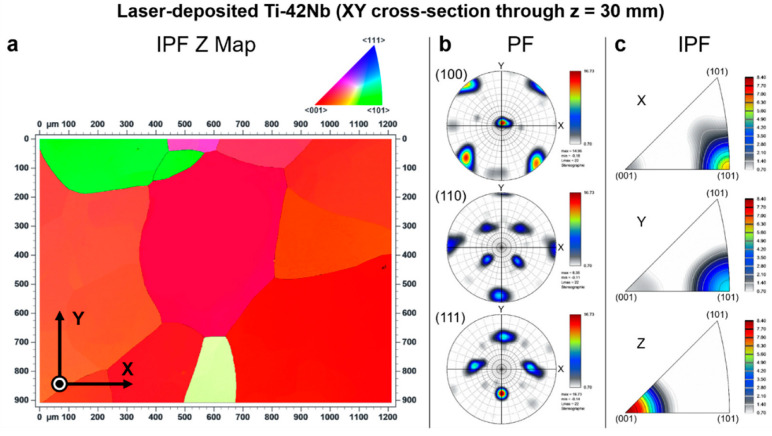
Electron backscatter diffraction (EBSD) results at XY cross-section through z = 30 mm: (**a**) inverse pole figure Z map (IPF Z Map), (**b**) pole figures (PF), and (**c**) inverse pole figures (IPF).

**Figure 10:**
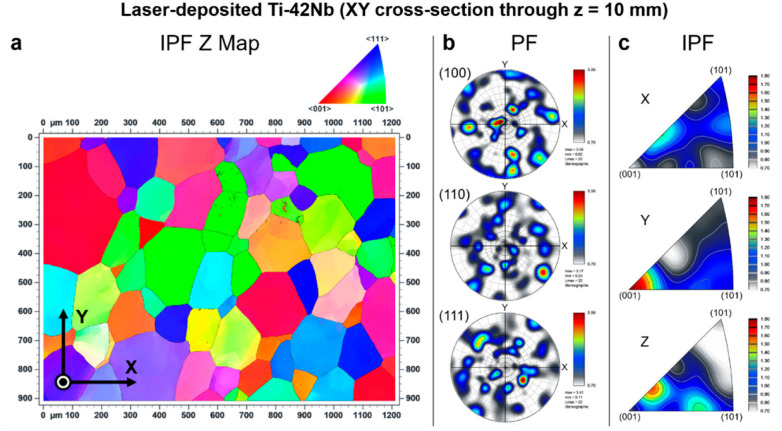
Electron backscatter diffraction (EBSD) results at XY cross-section through z = 10 mm: (**a**) inverse pole figure Z map (IPF Z Map), (**b**) pole figures (PF), and (**c**) inverse pole figures (IPF).

**Figure 11:**
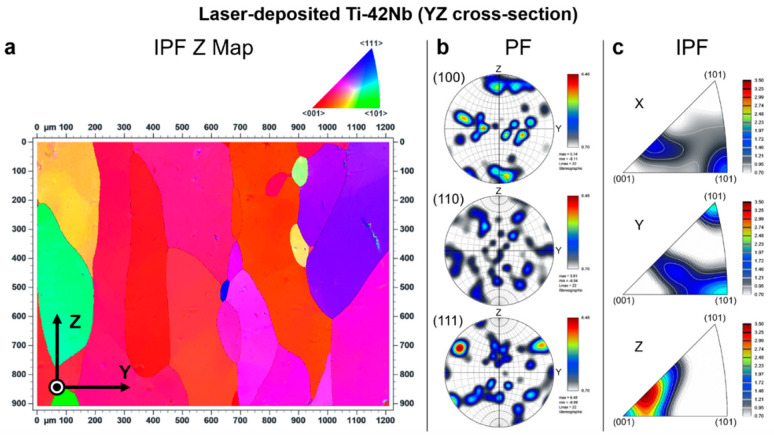
Electron backscatter diffraction (EBSD) results at YZ cross-section: (**a**) inverse pole figure Z map (IPF Z Map), (**b**) pole figures (PF), and (**c**) inverse pole figures (IPF).

Electron backscatter diffraction (EBSD) was carried out to analyze the crystallographic texture of the laser-deposited Ti-42Nb on the same samples analyzed by SEM and XRD. Results are shown in Figure 8, Figure 9, Figure 10 and Figure 11. Coarser grains and more intense texture were observed on the top of the specimen, at z = 50 mm and 30 mm (Figure 8 and Figure 9, respectively) than on the bottom, at z = 10 mm (Figure 10). In Figure 11, it can be seen that laser-deposited Ti-42Nb samples were composed of large elongated columnar grains aligned nearly parallel to the building direction (Z-axis) or normal to the substrate as a result of epitaxial growth from the parent grains. Laser-deposited Ti-42Nb samples presented a relatively intense <100> fiber texture, with a considerably great quantity of β-phase grains aligning one of their <100> axes nearly parallel to the building direction (Z-axis). This texture was particularly intense on the top of the sample (at z = 50 mm and 30 mm, Figure 8 and Figure 9, respectively).

The <100> fiber texture in β type Ti-42Nb alloy was developed after depositing several layers of material by LDED, even when the substrate was a cp-Ti grade 2 plate and not a seed crystal. These results suggest that parent grains with a favorable crystallographic orientation (<100> direction aligned with the building direction) grow faster during the LDED process; therefore, these favorably oriented grains become bigger and more predominant. Moreover, it seems that the growth of those grains with <110> direction nearly parallel with the scanning direction (X-axis and Y-axis) is favored as can be seen on the top of the specimen, at z = 50 mm and 30 mm (Figure 8 and Figure 9, respectively).

**Figure 12:**
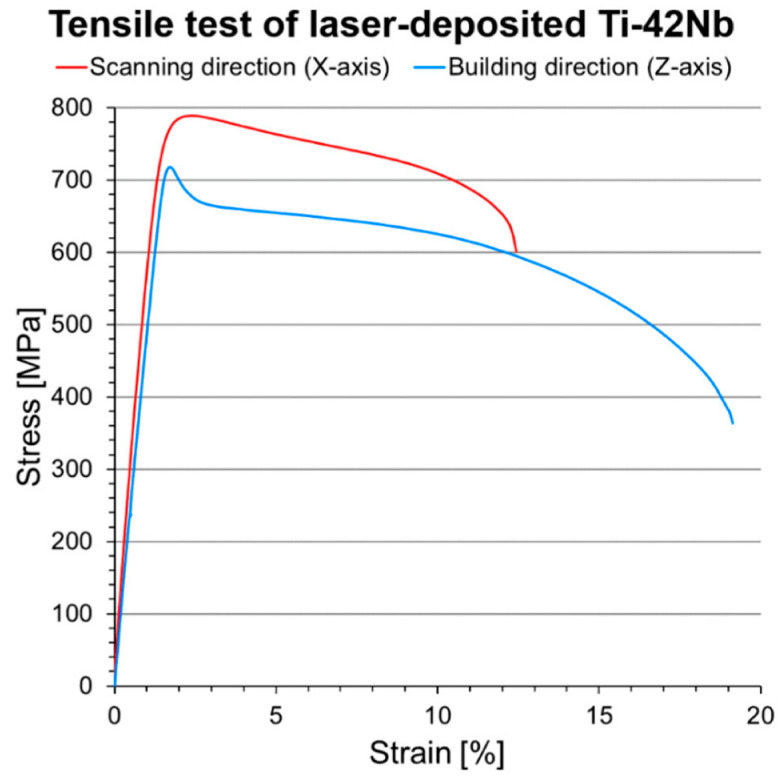
Representative engineering stress-strain curves of laser-deposited Ti-42Nb specimens in the scanning direction (X-axis) and building direction (Z-axis).

The representative engineering stress–strain curve from the tensile test of the laser-deposited Ti-42Nb specimens is shown in Figure 12. These laser-deposited ingots exhibited anisotropic mechanical properties because of their intense <100> fiber texture. The corresponding mechanical properties are tabulated in Table 1 and compared with selected biomedical titanium alloys. Laser-deposited Ti-42Nb possessed a relatively low modulus in the scanning direction (X-axis), E_x_ = 59.4 ± 3.0 GPa, but reasonably high yield strength (σ_0.2x_ = 735 ± 22 MPa) and tensile strength (UTS_x_ = 771 ± 22 MPa). Nevertheless, the elastic modulus of laser-deposited Ti-42Nb in the building direction (Z-axis) was even lower, E_z_ = 47.9 ± 3.9 GPa, without a significant decrease in yield (σ_0.2z_ = 715 ± 41 MPa) and tensile (UTS_z_ = 718 ± 42 MPa) strengths. These mechanical properties in the building direction were not significantly different from those obtained in the vertical direction (Z-axis) of Ti-42Nb generated by LPBF employing an unconventional top hat laser configuration [30].

The Owens, Wendt, Rabel, and Kaelble (OWRK) method was employed to calculate surface free energy and the dispersive and polar components from the contact angle (Table 2). There were no significant divergences between the contact angle of the laser-deposited Ti-42Nb samples and the cp-Ti grade 2 control.

**Figure 13:**
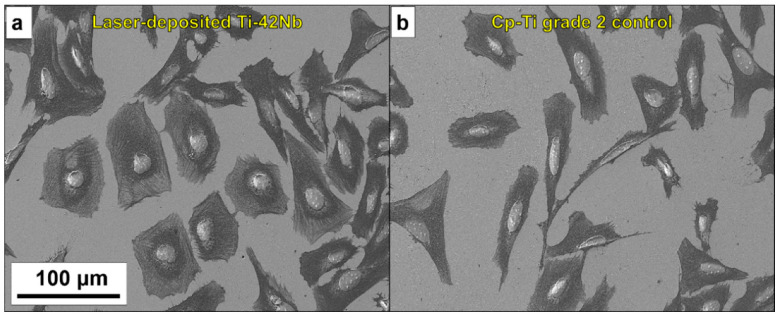
Micrographs showing cell adhesion: (**a**) laser-deposited Ti-42Nb, and (**b**) cp-Ti grade 2.

**Figure 14:**
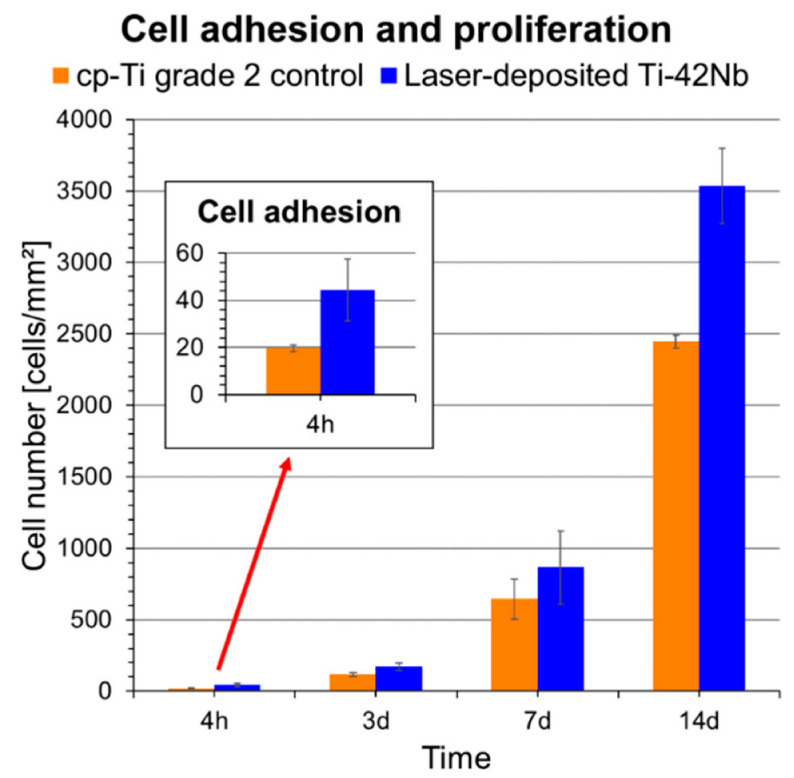
Cell adhesion and proliferation on laser-deposited Ti-42Nb samples and cp-Ti grade 2. The inset graphic shows the adhesion results. Error bars represent standard deviation.

The in vitro cytocompatibility was evaluated employing human osteoblast-like SaOS-2 cells. Laser-deposited Ti-42Nb alloy and cp-Ti grade 2 control were compared (Figure 13). The cell count showed significant dissimilarities in cell adhesion after 4 h of incubation (Figure 14), with the Ti-42Nb substrate being more favorable. Cells proliferated on both substrates, but the laser-deposited Ti-42Nb surface promoted their proliferation after 14 days of incubation. Although there were no significant differences between the areas of cell grown in both materials, the cell area was greater in the laser-deposited Ti-42Nb samples (Table 3). Laser-deposited Ti-42Nb alloy enhanced the osteoinductive effect, promoting the adhesion, proliferation, and spreading of SaOS-2 cells.

## 4. Discussion

These results indicate that laser-deposited β type Ti-42Nb alloy is a potentially promising candidate for the manufacturing of pioneering biomedical implants with a very low elastic modulus that can suppress stress shielding. The very low elastic modulus in the building direction (Z-axis) is due to the intense <100> fiber texture, with a considerably great quantity of grains aligning one of their <100> axes in that building direction (Z-axis). Tane et al. [21], in 2008, and Hermann et al. [20], in 2012, had already proposed that biocompatible β type titanium alloy single crystals whose {100} direction is oriented, or polycrystals with a texture in which the crystallographic <100> directions are oriented along a loading direction in human bones, could be candidates for biomedical implants with a low elastic modulus. However, the challenge is the development of new processes for manufacturing a large single-crystal or a textured polycrystal ingot and then accurately machining the biomedical implant.

In this paper, we have demonstrated the feasibility of laser-directed energy deposition (LDED) for the generation of a β type Ti-42Nb alloy textured polycrystal ingot with a very low modulus for the development of pioneering biomedical implants. The mechanical properties obtained by LDED are not significantly different to those obtained by the alternative additive manufacturing technique laser powder bed fusion (LPBF). Nevertheless, LDED processes have the capacity for producing larger components faster than SLM because they allow relatively higher deposition rates [31,54,55]. In addition, laser-deposited Ti-42Nb alloy is a more favorable substrate than cp-Ti grade 2 to promote the adhesion, proliferation, and spreading of SaOS-2 cells, which may be related to the higher cytocompatibility of Nb [8,56].

It is important to note, however, that more research is still needed, and additional tests are required to validate the performance of the laser-deposited β type Ti-42Nb alloy (e.g., fatigue testing, in vivo assessment, clinical studies). Moreover, there is still room to improve the process in order to intensify the crystallographic texture of the laser-deposited β type Ti-42Nb alloy and to enhance the anisotropy in the elastic modulus. For example, a more intense crystallographic texture could be developed by employing a seed crystal as a substrate, and, consequently, a lower elastic modulus could be achieved.

## 5. Conclusions

Biocompatible β type Ti-42Nb (wt%) alloy ingots were successfully generated by means of laser-directed energy deposition (LDED). Laser-deposited Ti-42Nb samples are composed of large elongated columnar grains aligned nearly normal to the substrate or parallel to the building direction (Z-axis) as a result of epitaxial growth from the parent grains. Moreover, an intense <100> fiber texture is observed, with a considerably great quantity of β-phase grains aligning one of their <100> axes nearly parallel to the building direction (Z-axis). The results suggest that parent grains with a favorable crystallographic orientation (<100> direction aligned with the building direction) grow faster during the LDED process; hence, these grains become bigger and more predominant. This intense <100> fiber texture in β type Ti-42Nb alloy is developed after depositing several layers of material by LDED, even when the substrate is a cp-Ti grade 2 plate and not a seed crystal. Laser-deposited Ti-42Nb specimens exhibit anisotropic mechanical properties because of their intense <100> fiber texture. In addition, we demonstrate that they possess a very low elastic modulus in the building direction (E_z_ = 47.9 ± 3.9 GPa) and high yield (σ_0.2z_ = 715 ± 41 MPa) and tensile (UTS_z_ = 718 ± 42 MPa) strengths. Laser-deposited Ti-42Nb alloy enhances the osteoinductive effect, promoting the adhesion, proliferation, and spreading of SaOS-2 cells. These results indicate that laser-deposited β type Ti-42Nb alloy is a potentially promising candidate for the manufacturing of pioneering biomedical implants with a very low elastic modulus that can suppress stress shielding.

## Figures and Tables

**Table 1:** Mechanical properties of selected biomedical titanium alloys determined in tensile tests: elastic modulus (E), yield strength (σ_0.2_), ultimate tensile strength (UTS), and elongation at break (ε_b_).

Material	Manufacturing	E [GPa]	σ_0.2_ [MPa]	UTS [MPa]	ε_b_ [%]
cp-Ti grade 2 [7]	–	102.7	275	345	20
cp-Ti grade 4 [7]	–	104.1	485	550	15
Ti-6Al-4V [7]	annealed	110–114	825–869	895–930	6–10
Ti-6Al-4V ELI [7]	mill annealed	101–110	795–875	860–965	10–15
Ti-45Nb [17]	annealed	64.3	438	527	–
Ti-42Nb [53]	LBPF-SLM	60.5 ± 4.0	674 ± 25	683 ± 17	11.7 ± 2.1
Ti-42Nb [30]	LPBF (X-axis)	51.0 ± 2.0	690 ± 12	720 ± 20	–
Ti-42Nb [30]	LPBF (Z-axis)	44.0 ± 2.0	674 ± 20	676 ± 21	–
Ti-27.5Nb (at%) [45]	LDED-CLAD^®^	70.0 ± 3.0	800 ± 5	820 ± 5	10
Ti-42Nb (present study)	LDED (X-axis)	59.4 ± 3.0	735 ± 22	771 ± 22	11.6 ± 4.0
Ti-42Nb (present study)	LDED (Z-axis)	47.9 ± 3.9	715 ± 41	718 ± 42	17.8 ± 1.4

**Table 2:** Surface free energy of samples (γ), dispersive component (γ_d_), polar component (γ_p_), contact angle with water (α_W_), and contact angle with diiodomethane (α_MI_).

Material	γ [mJ/m^2^]	γ_d_ [mJ/m^2^]	γ_p_ [mJ/m^2^]	α_W_ [°]	α_MI_ [°]
cp-Ti grade 2 (Control)	42.8 ± 3.2	36.8 ± 2.7	6.0 ± 2.4	75.4 ± 5.4	45.3 ± 5.2
Laser-deposited Ti-42Nb	42.7 ± 2.5	35.1 ± 1.0	7.6 ± 2.2	72.8 ± 4.8	48.5 ± 1.7

**Table 3:** Measured cell area.

Material	Cell Area [µm^2^]
cp-Ti grade 2 (Control)	450 ± 100
Laser-deposited Ti-42Nb	600 ± 200

## Data Availability

Not applicable.

## References

[1] Niinomi M., Nakai M., Hieda J. (2012). Development of new metallic alloys for biomedical applications. Acta Biomater..

[2] Szczęsny G., Kopec M., Politis D.J., Kowalewski Z.L., Łazarski A., Szolc T. (2022). A Review on Biomaterials for Orthopaedic Surgery and Traumatology: From Past to Present. Materials.

[3] Li Y., Yang C., Zhao H., Qu S., Li X., Li Y. (2014). New Developments of Ti-Based Alloys for Biomedical Applications. Materials.

[4] Kaur M., Singh K. (2019). Review on titanium and titanium based alloys as biomaterials for orthopaedic applications. Mater. Sci. Eng. C.

[5] Long M., Rack H.J. (1998). Titanium alloys in total joint replacement--a materials science perspective. Biomaterials.

[6] Zhang L., Chen L. (2019). A Review on Biomedical Titanium Alloys: Recent Progress and Prospect. Adv. Eng. Mater..

[7] Niinomi M. (1998). Mechanical properties of biomedical titanium alloys. Mater. Sci. Eng. A.

[8] Biesiekierski A., Wang J., Abdel-Hady Gepreel M., Wen C. (2012). A new look at biomedical Ti-based shape memory alloys. Acta Biomater..

[9] Kim K.M., Kim H.Y., Miyazaki S. (2020). Effect of Zr Content on Phase Stability, Deformation Behavior, and Young’s Modulus in Ti–Nb–Zr Alloys. Materials.

[10] Brizuela A., Herrero-Climent M., Rios-Carrasco E., Rios-Santos J., Pérez R., Manero J., Gil Mur J. (2019). Influence of the Elastic Modulus on the Osseointegration of Dental Implants. Materials.

[11] Mohammed M.T., Zahid A.K., Arshad N.S. (2014). Beta Titanium Alloys: The Lowest Elastic Modulus for Biomedical Applications: A Review. Int. J. Mater. Metall. Eng..

[12] Kolli R., Devaraj A. (2018). A Review of Metastable Beta Titanium Alloys. Metals.

[13] Kim H.Y., Hashimoto S., Kim J.I., Hosoda H., Miyazaki S. (2004). Mechanical properties and shape memory behavior of Ti-Nb alloys. Mater. Trans..

[14] Godley R., Starosvetsky D., Gotman I. (2006). Corrosion behavior of a low modulus β-Ti-45%Nb alloy for use in medical implants. J. Mater. Sci. Mater. Med..

[15] Gostin P.F., Helth A., Voss A., Sueptitz R., Calin M., Eckert J., Gebert A. (2013). Surface treatment, corrosion behavior, and apatite-forming ability of ti-45Nb implant alloy. J. Biomed. Mater. Res.-Part B Appl. Biomater..

[16] Karre R., Niranjan M.K., Dey S.R. (2015). First principles theoretical investigations of low Young’s modulus beta Ti-Nb and Ti-Nb-Zr alloys compositions for biomedical applications. Mater. Sci. Eng. C.

[17] Bai Y., Deng Y., Zheng Y., Li Y., Zhang R., Lv Y., Zhao Q., Wei S. (2016). Characterization, corrosion behavior, cellular response and in vivo bone tissue compatibility of titanium-niobium alloy with low Young’s modulus. Mater. Sci. Eng. C.

[18] Markhoff J., Weinmann M., Schulze C., Bader R. (2017). Influence of different grained powders and pellets made of Niobium and Ti-42Nb on human cell viability. Mater. Sci. Eng. C.

[19] Wang J., Xiao W., Ren L., Fu Y., Ma C. (2021). The roles of oxygen content on microstructural transformation, mechanical properties and corrosion resistance of Ti-Nb-based biomedical alloys with different β stabilities. Mater. Charact..

[20] Hanada S., Matsumoto H., Watanabe S. (2005). Mechanical compatibility of titanium implants in hard tissues. Int. Congr. Ser..

[21] Ozaki T., Matsumoto H., Watanabe S., Hanada S. (2004). Beta Ti Alloys with Low Young’s Modulus. Mater. Trans..

[22] Hermann R., Hermann H., Calin M., Büchner B., Eckert J. (2012). Elastic constants of single crystalline β-Ti_70_Nb_30_. Scr. Mater..

[23] Tane M., Akita S., Nakano T., Hagihara K., Umakoshi Y., Niinomi M., Nakajima H. (2008). Peculiar elastic behavior of Ti–Nb–Ta–Zr single crystals. Acta Mater..

[24] Takesue N., Shimizu Y., Yano T., Hara M., Kuramoto S. (2009). Single-crystal growth of Ti–Nb–Ta–Zr–O alloys and measurement of elastic properties. J. Cryst. Growth.

[25] Zhang Y.W., Li S.J., Obbard E.G., Wang H., Wang S.C., Hao Y.L., Yang R. (2011). Elastic properties of Ti–24Nb–4Zr–8Sn single crystals with bcc crystal structure. Acta Mater..

[26] Lee S.-H., Todai M., Tane M., Hagihara K., Nakajima H., Nakano T. (2012). Biocompatible low Young’s modulus achieved by strong crystallographic elastic anisotropy in Ti–15Mo–5Zr–3Al alloy single crystal. J. Mech. Behav. Biomed. Mater..

[27] Wang P., Todai M., Nakano T. (2019). Beta titanium single crystal with bone-like elastic modulus and large crystallographic elastic anisotropy. J. Alloys Compd..

[28] Ishimoto T., Hagihara K., Hisamoto K., Sun S.H., Nakano T. (2017). Crystallographic texture control of beta-type Ti–15Mo–5Zr–3Al alloy by selective laser melting for the development of novel implants with a biocompatible low Young’s modulus. Scr. Mater..

[29] Shinohara Y., Matsumoto Y., Tahara M., Hosoda H., Inamura T. (2018). Development of <001>-fiber texture in cold-groove-rolled Ti-Mo-Al-Zr biomedical alloy. Materialia.

[30] Pilz S., Gustmann T., Günther F., Zimmermann M., Kühn U., Gebert A. (2022). Controlling the Young’s modulus of a ß-type Ti-Nb alloy via strong texturing by LPBF. Mater. Des..

[31] Arias-González F., Barro O., del Val J., Lusquiños F., Fernández-Arias M., Comesaña R., Riveiro A., Pou J. (2021). Laser-directed energy deposition. Additive Manufacturing.

[32] Saboori A., Gallo D., Biamino S., Fino P., Lombardi M. (2017). An Overview of Additive Manufacturing of Titanium Components by Directed Energy Deposition: Microstructure and Mechanical Properties. Appl. Sci..

[33] Zhao Z., Wang C., Yu Q., Song L., Yang G., Zhang J. (2022). New β-type Ti-Zr-V-Nb alloys used for laser-based direct energy deposition: Design, microstructure, and properties. Mater. Charact..

[34] Arias-González F., del Val J., Comesaña R., Penide J., Lusquiños F., Quintero F., Riveiro A., Boutinguiza M., Gil F.J., Pou J. (2018). Microstructure and crystallographic texture of pure titanium parts generated by laser additive manufacturing. Met. Mater. Int..

[35] Barro Ó., Arias-González F., Lusquiños F., Comesaña R., Del Val J., Riveiro A., Badaoui A., Gómez-Baño F., Pou J. (2020). Effect of four manufacturing techniques (Casting, laser directed energy deposition, milling and selective laser melting) on microstructural, mechanical and electrochemical properties of co-cr dental alloys, before and after pfm firing process. Metals.

[36] Barro Ó., Arias-González F., Lusquiños F., Comesaña R., Del Val J., Riveiro A., Badaoui A., Gómez-Baño F., Pou J. (2021). Improved commercially pure titanium obtained by laser directed energy deposition for dental prosthetic applications. Metals.

[37] Arias-González F., Rodríguez-Contreras A., Punset M., Manero J.M., Barro Ó., Fernández-Arias M., Lusquiños F., Gil F.J., Pou J. (2021). In-Situ Laser Directed Energy Deposition of Biomedical Ti-Nb and Ti-Zr-Nb Alloys from Elemental Powders. Metals.

[38] Comesaña R., Lusquiños F., del Val J., López-Álvarez M., Quintero F., Riveiro A., Boutinguiza M., de Carlos A., Jones J.R., Hill R.G. (2011). Three-dimensional bioactive glass implants fabricated by rapid prototyping based on CO_2_ laser cladding. Acta Biomater..

[39] Comesaña R., Lusquiños F., del Val J., Malot T., López-Álvarez M., Riveiro A., Quintero F., Boutinguiza M., Aubry P., De Carlos A. (2011). Calcium phosphate grafts produced by rapid prototyping based on laser cladding. J. Eur. Ceram. Soc..

[40] Lusquiños F., del Val J., Arias-González F., Comesaña R., Quintero F., Riveiro A., Boutinguiza M., Jones J.R., Hill R.G., Pou J. (2014). Bioceramic 3D Implants Produced by Laser Assisted Additive Manufacturing. Phys. Procedia.

[41] Comesaña R., Lusquiños F., del Val J., Quintero F., Riveiro A., Boutinguiza M., Jones J.R., Hill R.G., Pou J. (2015). Toward Smart Implant Synthesis: Bonding Bioceramics of Different Resorbability to Match Bone Growth Rates. Sci. Rep..

[42] Xue W., Krishna B.V., Bandyopadhyay A., Bose S. (2007). Processing and biocompatibility evaluation of laser processed porous titanium. Acta Biomater..

[43] Krishna B.V., Bose S., Bandyopadhyay A. (2007). Low stiffness porous Ti structures for load-bearing implants. Acta Biomater..

[44] Dinda G.P., Song L., Mazumder J. (2008). Fabrication of Ti-6Al-4V scaffolds by direct metal deposition. Metall. Mater. Trans. A Phys. Metall. Mater. Sci..

[45] Fischer M., Laheurte P., Acquier P., Joguet D., Peltier L., Petithory T., Anselme K., Mille P. (2017). Synthesis and characterization of Ti-27.5Nb alloy made by CLAD^®^ additive manufacturing process for biomedical applications. Mater. Sci. Eng. C.

[46] Wei J., Sun H., Zhang D., Gong L., Lin J., Wen C. (2018). Influence of Heat Treatments on Microstructure and Mechanical Properties of Ti–26Nb Alloy Elaborated In Situ by Laser Additive Manufacturing with Ti and Nb Mixed Powder. Materials.

[47] Kalita D., Rogal Ł., Bobrowski P., Durejko T., Czujko T., Antolak-Dudka A., Cesari E., Dutkiewicz J. (2020). Superelastic Behavior of Ti-Nb Alloys Obtained by the Laser Engineered Net Shaping (LENS) Technique. Materials.

[48] Banerjee R., Nag S., Samuel S., Fraser H.L. (2006). Laser-deposited Ti-Nb-Zr-Ta orthopedic alloys. J. Biomed. Mater. Res.-Part A.

[49] Samuel S., Nag S., Nasrazadani S., Ukirde V., El Bouanani M., Mohandas A., Nguyen K., Banerjee R. (2010). Corrosion resistance and in vitro response of laser-deposited Ti-Nb-Zr-Ta alloys for orthopedic implant applications. J. Biomed. Mater. Res.-Part A.

[50] Nag S., Banerjee R. (2012). Laser deposition and deformation behavior of Ti–Nb–Zr–Ta alloys for orthopedic implants. J. Mech. Behav. Biomed. Mater..

[51] Beausir B., Fundenberger J.J. (2017). Analysis Tools for Electron and X-ray Diffraction, ATEX–Software.

[52] (2019). Metallic Materials—Tensile Testing—Part 1: Method of Test at Room Temperature.

[53] Schulze C., Weinmann M., Schweigel C., Keßler O., Bader R. (2018). Mechanical Properties of a Newly Additive Manufactured Implant Material Based on Ti-42Nb. Materials.

[54] DebRoy T., Wei H.L., Zuback J.S., Mukherjee T., Elmer J.W., Milewski J.O., Beese A.M., Wilson-Heid A., De A., Zhang W. (2018). Additive manufacturing of metallic components–Process, structure and properties. Prog. Mater. Sci..

[55] Karunakaran K.P., Bernard A., Suryakumar S., Dembinski L., Taillandier G. (2012). Rapid manufacturing of metallic objects. Rapid Prototyp. J..

[56] Yamamoto A., Honma R., Sumita M. (1998). Cytotoxicity evaluation of 43 metal salts using murine fibroblasts and osteoblastic cells. J. Biomed. Mater. Res..

